# A sound approach to advancing healthcare systems: the future of biomedical acoustics

**DOI:** 10.1038/s41467-022-31014-y

**Published:** 2022-06-16

**Authors:** Joseph Rufo, Peiran Zhang, Ruoyu Zhong, Luke P. Lee, Tony Jun Huang

**Affiliations:** 1 Department of Mechanical Engineering and Materials Science, Duke University, Durham, NC 27708 USA; 2 Renal Division and Division of Engineering in Medicine, Department of Medicine, Brigham and Women’s Hospital, Harvard Medical School, Boston, MA 02115 USA

**Keywords:** Lab-on-a-chip, Biomedical engineering, Acoustics, Microfluidics

## Abstract

Newly developed acoustic technologies are playing a transformational role in life science and biomedical applications ranging from the activation and inactivation of mechanosensitive ion channels for fundamental physiological processes to the development of contact-free, precise biofabrication protocols for tissue engineering and large-scale manufacturing of organoids. Here, we provide our perspective on the development of future acoustic technologies and their promise in addressing critical challenges in biomedicine.

## Abstract (web summary)

Acoustic techniques are moving towards the clinic. Here the authors highlight recent developments in the areas of acoustic mechanobiology, point-of-care diagnostics, in vivo manipulation and tissues engineering, and provide their thoughts on the current challenges and directions for future work.

## Introduction

**Fig. 1:**
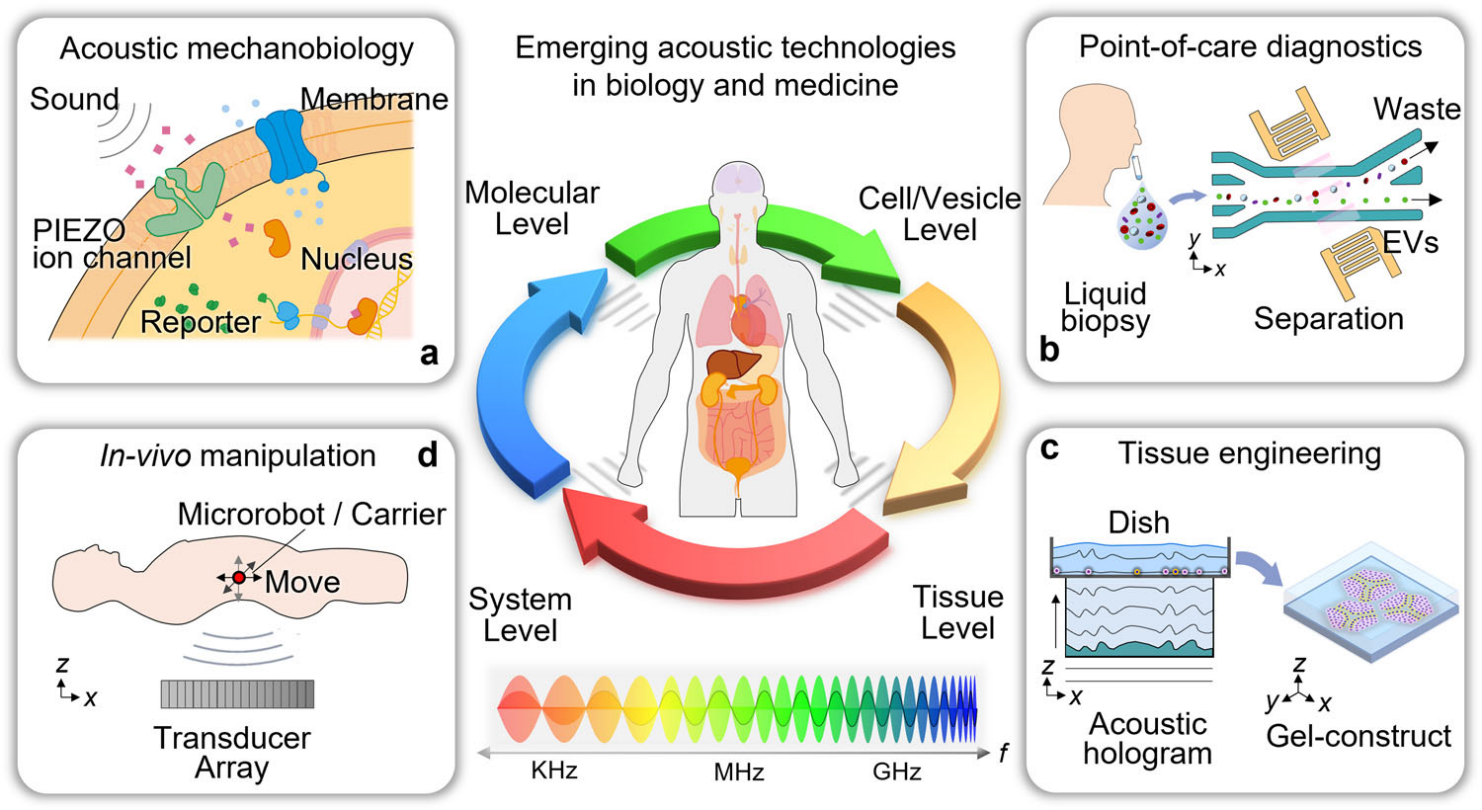
The future of biomedical acoustics. An overview of the four emerging areas of research in developing acoustic technologies for use in biology and medicine including **a** acoustic mechanobiology, **b** point-of-care diagnostics, **c** biofabrication and tissue engineering, and **d** in vivo acoustic manipulation. Acoustic technologies utilize frequencies spanning the kHz to GHz range, enabling them to manipulate nanometer-sized objects, such as ion channels, at the molecular level and millimeter-sized objects, such as microrobots, at the system level. EVs: extracellular vesicles.

The use of sound has a long history in medicine. Dating back to 350 BC, the ancient Greek physician Hippocrates, regarded as “the father of medicine,” devised a diagnostic method for detecting fluid in the lungs by shaking patients by their shoulders and listening to the resulting sounds emanating from their chest^1^. As acoustic technology has advanced, so too has our ability to “listen” to the body and better understand underlying pathologies. The 19th-century invention of the stethoscope allowed doctors to gauge the health of the heart^2^; the 20th-century invention of ultrasound imaging revolutionized the field of biomedical imaging and enabled doctors to diagnose a range of conditions in the fields of obstetrics, emergency medicine, and cardiology^3^; the 21st-century inventions of photoacoustic imaging^4^ and photoacoustic microscopy^5^ have led to significant advances in the ability to perform functional imaging, enabling doctors to not only visualize organs and tissues at high resolution but also gain insights into underlying biochemical activity. While acoustics has become commonplace for many medical applications, several potentially transformative acoustic technologies have been developed over the last decade. These exciting laboratory demonstrations are beginning to show their potential in clinical applications. Here, we will review recent developments in acoustic technologies in four emerging areas of research (Fig. 1), provide our perspective on the challenges that need to be addressed, and share our vision of the future applications that these newly developed acoustic technologies will enable.

### From basic acoustic mechanobiology to non-invasive acoustic therapeutics

**Fig. 2:**
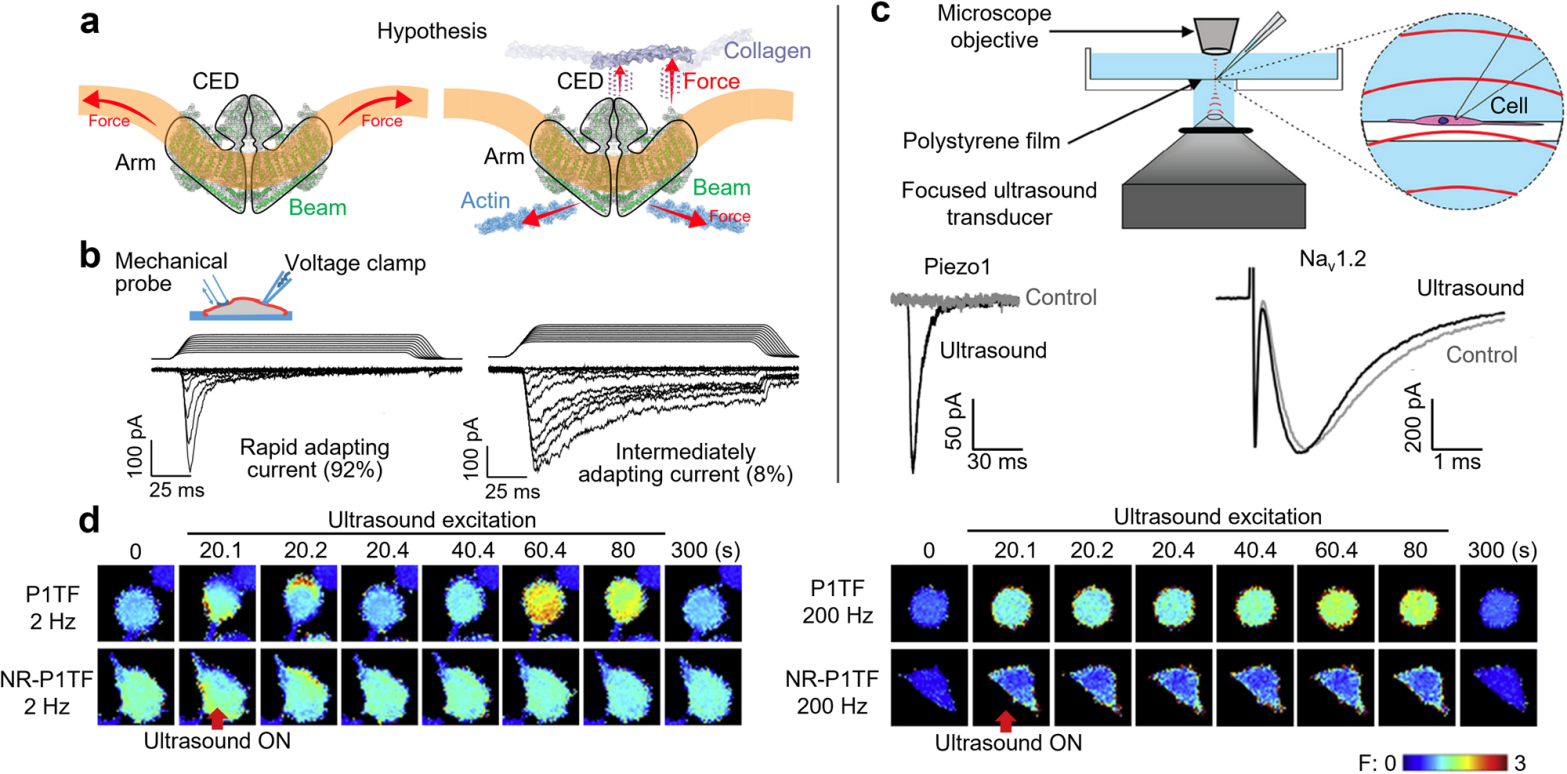
Acoustic technologies for mechanobiology. Acoustic technologies are being utilized to study mechanically sensitive ion channels, such as Piezo1 and Piezo2. **a** Two models have been proposed for activation of the Piezo1 channel: the lateral membrane tension model (left), in which tension in the membrane leads to a gating force, and the tethered spring model (right), in which the activation of the channel is governed by interactions with the extracellular matrix. **b** Representative traces showing how traditional mechanical activation is used to study the dynamics of mechanosensitive neurons. A force is applied to the cell membrane using a glass probe, while another pipette is used to perform simultaneous patch-clamp current measurements in a whole-cell configuration. 92% of the neurons studied responded with a rapidly adapting current (inactivation time <10 ms), while 8% responded with an intermediately adapting current (10 ms < inactivation time <30 ms). **c** Ultrasound can be used to activate the mechanosensitive Piezo1 channel and the voltage-gated sodium channel NaV1.2 and make simultaneous patch-clamp recordings. **d** Ultrasound can also be used to target Piezo1-transfected (P1TF) HEK293T cells and investigate the intracellular calcium response under different pulse conditions. Fluorescence images show the intracellular calcium response of both responsive P1TF cells and non-responsive P1TF cells (NR-P1TF) under different pulse repetition frequencies. Part **a** adapted with permission from ref. ^7^, Springer Nature. Part **b** adapted with permission from ref. ^8^, Springer Nature. Part **c** adapted with permission from ref. ^11^, Elsevier. Part **d** adapted with permission from ref. ^12^, Elsevier.

Mechanical stimuli send signals not only for many physiological processes but also for molecular and cellular level actuations. Recently, Ardem Patapoutian was awarded the 2021 Nobel Prize in Physiology or Medicine for his discovery of the mechanically sensitive ion channels Piezo1 and Piezo2^6^, which are force-sensing integral membrane proteins expressed in many mechanosensitive cell types (Fig. 2a)^7^. A traditional method for measuring the response of mechanosensitive cells utilizes glass probes to apply well-defined forces to the cell membrane, while another pipette is used to perform simultaneous patch-clamp current measurements in a whole-cell configuration (Fig. 2b)^8^. However, the potential use of focused ultrasound to activate ion channels, such as Piezo1 and Piezo2, opens new opportunities in fundamental life science and translational medicine to study the role of mechanotransduction in touch, pain, proprioception, hearing, blood flow, flow sensing in the kidney, and embryonic development^9^. Compared to other modulation methods, such as optogenetics, which has a limited penetration depth due to the rapid absorption and scattering of light in biological tissues and requires invasive permanent gene modification of ion channels, natural ultrasound activation provides a few advantages^10^. First, it offers a non-invasive method to transmit energy through complex boundaries, such as the skull. Second, it allows us to focus on and target deep regions within the brain. Third, ultrasound can exert molecular level control over neural activity without permanent gene modifications. Recently, Prieto et al. reported a selective activation of the mechanosensitive ion channels Piezo1, but not Na_v_1.2 channels, using ultrasound at 43 MHz (Fig. 2c)^11^. Liao et al. used an alternative device setup to demonstrate the activation of the Piezo1 using ultrasound at 30 MHz, while simultaneously investigating the impact of the pulse repetition frequency (Fig. 2d)^12^. By generating innovative acoustic tools to modulate the electrical activity of excitable cells, we can obtain a deeper understanding of the mechanisms which govern mechanotransduction. In the coming years, might it be possible to image the opening of mechanosensitive ion channels in real-time, a feat that has yet to be accomplished? In addition to gaining new wisdom and insight on mechanosensitive ion channels, we expect advances in targeted acoustic technologies to activate or inhibit mechanosensitive ion channels of neurons, muscle, or cancer cells, as well as neuromuscular junctions, as a potential treatment for neurological disorders, neurodegenerative diseases, neuromuscular junction disorder, and cancer. The mechanosensitive channels also can function as sonogenetic actuators for future acoustic neuromodulation^13,14^ of targeted cells.

### Acoustofluidic point-of-care diagnostics

**Fig. 3:**
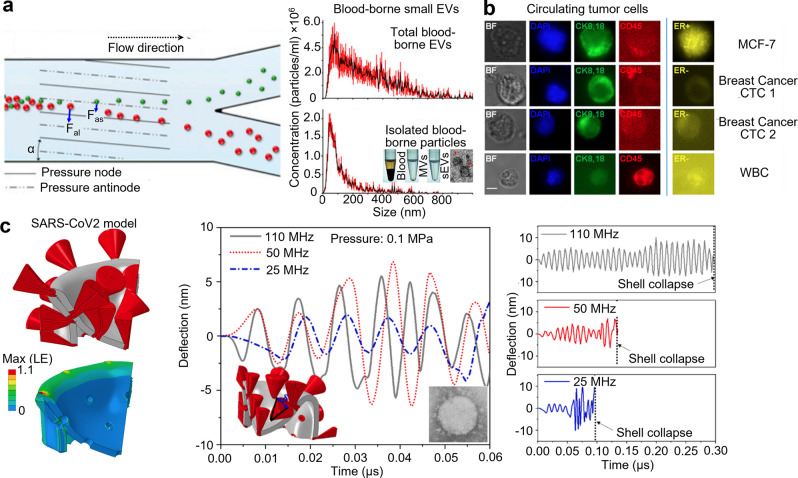
Acoustic technologies for point-of-care diagnostics. **a** Acoustic tweezers technologies have been developed to separate bioparticles based on differences in their physical properties. Differences in the size, density, and compressibility of bioparticles determine the magnitudes of the acoustic radiation forces that they will experience, enabling different bioparticles to be pushed to different outlets under continuous flow (left). Nanoparticle tracking analysis results show the size distribution of extracellular vesicles (EVs) found in human plasma and the small extracellular vesicles (sEVs) isolated by the acoustic tweezers device (right). **b** Acoustic tweezers devices have also been developed that can separate circulating tumor cells (CTCs) from white blood cells (WBCs). Immunofluorescence images of the isolated samples are used to verify the isolation of CTCs from WBCs. **c** Numerical modeling results show the vibration amplitude of the spike protein of the SARS-CoV-2 virus under ultrasound excitation. These simulation results suggest that ultrasound can be used to deflect the spikes and generate high strains in the virus shell, potentially leading to the collapse of the shell and destruction of the virus. Part **a** adapted with permission from ref. ^18^, National Academy of Science. Part **b** adapted with permission from ref. ^23^, National Academy of Science. Part **c** adapted with permission from ref. ^29^, Elsevier.

The contactless, biocompatible nature of acoustic waves, along with its versatility, excellent spatiotemporal resolution, and a high potential for customization (i.e., many acoustic parameters that can be tuned over a wide working region—operating frequencies from the kHz to GHz range), enable them to become a powerful platform for point-of-care diagnosis^15,16^. For example, multiple “acoustic tweezers” technologies have been developed to separate bioparticles based on minute differences in their physical properties (Fig. 3a)^17–23^. Early demonstrations showed the ability to isolate biomarkers such as circulating tumor cells from white blood cells^20^ and peripheral blood samples^23^, showing its potential in early cancer detection and diagnostics (Fig. 3b). However, in recent years, as the spatial resolution of acoustic tweezers technology has improved, many research efforts have focused on using acoustic tweezers to isolate nanometer-sized extracellular vesicles called small extracellular vesicles (sEVs) from various biofluids^18,24^.

sEVs contain DNA, miRNA, mRNA, and proteins from their cellular origin and are abundant in nearly all biofluids^25^. As a result, they have been identified as a promising target for developing liquid biopsies for non-invasive disease diagnostics and treatment monitoring for cancer^26^ and neurodegenerative diseases^27^. However, current sEV isolation technologies, such as ultracentrifugation, suffer from lengthy processing times, require highly trained operators, and are limited in their ability to isolate high-purity sEVs with high yield. As a result, the clinical utility of sEVs, especially in point-of-care applications, has been limited. Recently, innovative acoustic technologies have been developed to isolate sEVs from whole blood^18^, saliva^24^, and urine^28^ in an automated manner. These acoustic tweezers technologies showed significantly improved purity and yield compared with ultracentrifugation. These newly developed acoustic technologies for the isolation of sEVs show great promise for the development of sEV-based liquid biopsies. They may help to unlock the clinical potential of sEVs as a circulating biomarker.

Can acoustic tweezers technologies also be developed to isolate viruses from various biofluids based on the similar size of sEVs (30–150 nm) and viruses (SARS-CoV-2 diameter: 70–120 nm)? By isolating viruses from biofluids with high purity and high yield, acoustic tweezers will improve the detection limit of downstream viral detection technologies. As a result, it can enable earlier diagnoses of viral infections. Due to the point-of-care nature of acoustic tweezers devices, they can be easily and rapidly deployed, rendering them a potentially powerful tool to help combat future viral outbreaks. In addition to isolating viral particles, can acoustic tweezers technologies also find applications as a therapeutic tool for fighting viral infections via selective viral filtration or the selective destruction of viral particles? There is reason to explore the use of acoustic tweezers technologies in the selective destruction of viral particles; recent simulation results suggest that under applied ultrasound of 25–50 MHz at 0.1 MPa, the viral envelope of the SARS-CoV-2 virus will collapse in as little as 0.1 µs (Fig. 3c)^29^. These parameters are within the operating range of previously developed acoustic tweezers devices. In the coming years, we believe that a growing area of acoustic tweezers research will focus on developing techniques to isolate selectively and destroy viruses for diagnostic and therapeutic applications.

While much progress has been made in developing acoustofluidic point-of-care diagnostic technologies, the research community must focus on the use of mechanically robust materials. Most acoustic tweezers or acoustofluidic devices utilize polydimethylsiloxane (PDMS), which is an excellent material for rapid prototyping but very difficult to incorporate into commercial products. Developing large-scale manufacturable plastic-based chips for acoustic devices would greatly aid commercialization efforts. In addition, it is essential to transition from the current “chip-in-a-lab” approach to a true “lab-on-a-chip” approach. Most demonstrations of acoustic separation technologies require extensive external equipment, such as highly precise pumps, function generators, and amplifiers. In order to facilitate widespread adoption of acoustic separation technology, we must integrate these components into fully functional prototypes. There is also a need to incorporate acoustic technologies with detection strategies that are true “point-of-care.” Although many acoustic technologies can rapidly isolate bioparticles, these advantages are lost if samples must be analyzed by lengthy analytical procedures like a reverse transcription-polymerase chain reaction (RT-PCR) or traditional immunoassay like ELISA for molecular diagnostics. More focus should be placed on integrating sample preparation on-chip by acoustic technology and rapid analytical sensing platforms, such as ultrafast photonic PCR^30^ and/or immuno-PCR on-chip^31^, or immunoassays with electrochemical, plasmonic, colorimetric, and fluorescence-based readouts. We envision the best complementary team will create the ultrafast and ultra-sensitive label-free molecular diagnostic system-on-chip with integrated acoustofluidics and optical or electrical sensors.

### Biofabrication, tissue engineering, organs, and organoids on-chip

**Fig. 4:**
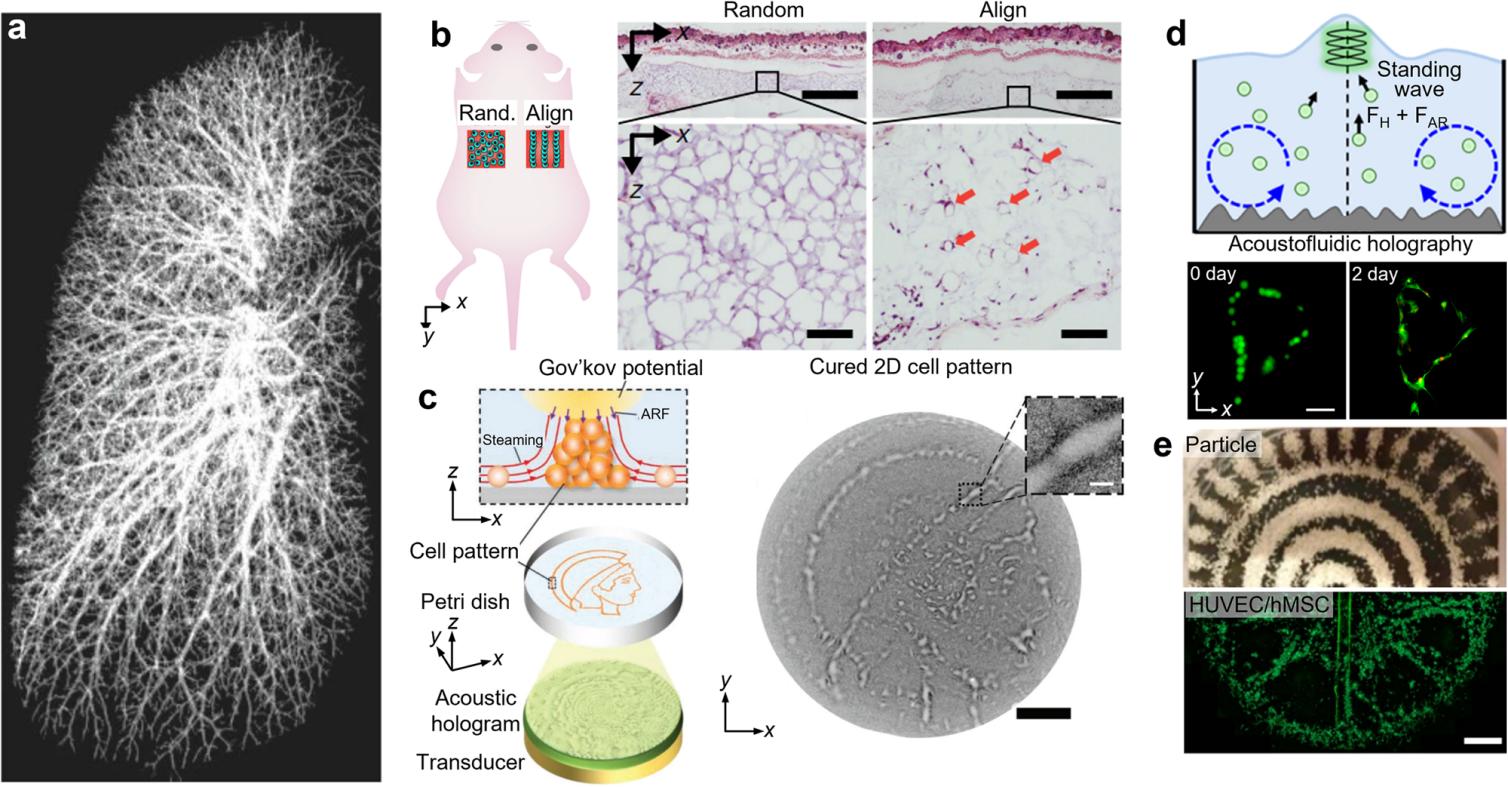
Acoustic technologies for tissue engineering. **a** Computed tomographic (CT) images showing the complexity of the arteries and veins found within human lungs. Recreating these intricate vascular trees remains a challenge for state-of-the-art tissue engineering techniques. **b** Acoustic waves were used to align human stem cells and endothelial cells into precise lines within a hydrogel. When transplanted into ischemic muscle in a mouse model of hindlimb ischemia, the acoustically aligned stem cells and endothelial cells exhibited an increased density of microvessels and enhanced paracrine effects as compared to randomly distributed cells. Scale bars: 500 μm (upper image) and 200 μm (lower image). **c** Acoustic holography represents one approach for fabricating complex, arbitrary patterns of cells in hydrogels, which then undergo gelation to immobilize the patterns. Scale bar: 5 mm. Scale bar (inset): 500 µm. **d** An acoustofluidic holography approach that also utilizes the induced fluid motion to pattern cells in hydrogels with higher spatial resolution. Scale bar: 50 µm. **e** An alternative acoustic approach known as sound-induced morphogenesis (SIM) can be used to rapidly pattern particles (top) and cells (bottom) in hydrogels. Here, the patterns formed are a function of the acoustic parameters and chamber geometry. Scale bar: 500 µm. Part **a** adapted from ref. ^38^, CC BY 4.0 (https://creativecommons.org/licenses/by/4.0/). Part **b** adapted from ref. ^40^, CC BY 4.0 (https://creativecommons.org/licenses/by/4.0/). Part **c** adapted from ref. ^41^, CC BY 4.0 (https://creativecommons.org/licenses/by/4.0/). Part **d** reprinted with permission from ref. ^42^. Copyright 2020 American Chemical Society. Part **e** adapted from ref. ^44^, CC BY 4.0 (https://creativecommons.org/licenses/by/4.0/).

Over the past two decades, research in the field of tissue engineering and organ and organoids on-chip has shown tremendous potential in disease treatment, drug testing, disease modeling, and enabled advanced studies of physiological and pathological processes^32,33^. Traditional approaches for biofabrication and tissue engineering, such as 3D bioprinting, have shown great promise for achieving the goal of “printing” functional organ replacements. Many different 3D bioprinting approaches, including laser-assisted^34^, stereolithography^35^, jetting-based^36^, and microextrusion-based^37^ bioprinting have been implemented. Each technique has its strengths and weaknesses; however, all 3D printing-based methods for tissue engineering suffer from a fundamental constraint: the inability to print at high-cell densities. While cell densities from 3D printing-based techniques can approach 10^8^ cells/mL, this is at least two orders of magnitude lower than the cell densities observed in vivo^38^. The limitation is primarily due to the difficulties of creating the perfusable vascular architectures required to supply oxygen and nutrients to densely packed cells (Fig. 4a). As a result, 3D printed tissues lack the necessary microstructural complexity to achieve physiologically relevant functions. In this regard, the development of a fabrication technique that can print high-density, vascularized tissues in a high-throughput and biocompatible manner would be of tremendous value to the field of tissue engineering.

Recently developed acoustic technologies provide innovative solutions to address these critical issues in 3D tissue engineering and enable the fabrication of high-cell-density, biomimetic tissue constructs. Rather than “printing” cells, acoustics can precisely position cells within hydrogels into the desired geometry. Recently, Armstrong et al. utilized acoustic waves to align myoblasts within hydrogels, enabling the formation of muscle tissues with aligned bundles of myotubes^39^. These acoustically aligned, high-density tissue structures better replicated the structure found in natural muscle tissue and exhibited anisotropic tensile mechanics. This demonstration was the first in vitro demonstration that aligned cellular fibers can be created using acoustic patterning, addressing a fundamental limitation in fabricating biomimetic muscle tissue. More recently, Kang et al. demonstrated the potential of acoustic technologies to fabricate vascularized tissue and showed its potential under in vivo conditions by transplanting the tissue into a mouse model (Fig. 4b)^40^. This approach utilizes acoustic waves coupled to a removable microfluidic chamber to align human stem cells and endothelial cells into precise lines within a hydrogel. The acoustic coupling enables the cells to be patterned in three dimensions, leading to the fabrication of tissue constructs that replicate vessels in skeletal muscle. When transplanted into ischemic muscle in a mouse model of hindlimb ischemia, the acoustically aligned stem cells and endothelial cells exhibited an increased density of microvessels and enhanced paracrine effects as compared to randomly distributed cells. These exciting results demonstrate the potential of acoustic technologies in generating functional tissue and pave the way for the use of acoustic technologies in therapeutic tissue engineering applications.

While the previously mentioned approaches represent exciting approaches for engineering biomimetic tissue structures, the alignment of cells in 2D and 3D has been limited to linear fiber-shaped structures. To enable the fabrication of more complex shapes and patterns, concepts from acoustic holography have recently been implemented in tissue engineering applications. For example, the acoustic holography technique developed by Ma et al. demonstrates the ability to create complex, arbitrary acoustic fields, which can be used to pattern cells within biocompatible hydrogels in a contact-free manner (Fig. 4c)^41^. This holographic approach solves a long-standing issue in using acoustics to pattern cells, which were previously based on standing waves to generate relatively simple geometric arrays such as lines, columns, or sheets. The ability to pattern cells into arbitrary, complex, three-dimensional shapes will enable researchers to fabricate tissue constructs that better mimic the natural state of their intended target tissue. More recently, another technology based on acoustic holography developed by Gu et al. was capable of patterning and concentrating cells within biocompatible hydrogels (Fig. 4d)^42^. The use of acoustic holograms to increase the local concentration of cells within hydrogels is one potential strategy to overcome the low cell densities that are typically observed when working with “cells in gels.”

In the coming decade, can these newly developed acoustic holography technologies be combined with existing 3D bioprinting technologies to enable the printing of functional tissue constructs? We believe that the marriage between these two powerful technologies is inevitable and will lead to next-generation 3D bioprinters. In addition, there is already precedent for the merger of acoustic technologies with 3D bioprinters. For example, Sriphutkiat et al. combined an acoustic nozzle with an extrusion 3D bioprinter to align cells as they were extruded from the nozzle and improve the cellular alignment, which resulted in enhanced proliferation and differentiation^43^. mimiX Biotherapeutics is in the process of commercializing the first acoustic bioprinter, which makes use of sound-induced morphogenesis (SIM) to pattern cells within a Petri dish-like container (Fig. 4e)^44^. This approach can be used to pattern cells into relatively simple tissue constructs such as spheroids and organoids. However, as more advanced acoustic technologies, such as acoustic holography, are integrated with various 3D bioprinting approaches, we expect to see rapid growth in the fabrication of biomimetic tissues.

**Fig. 5:**
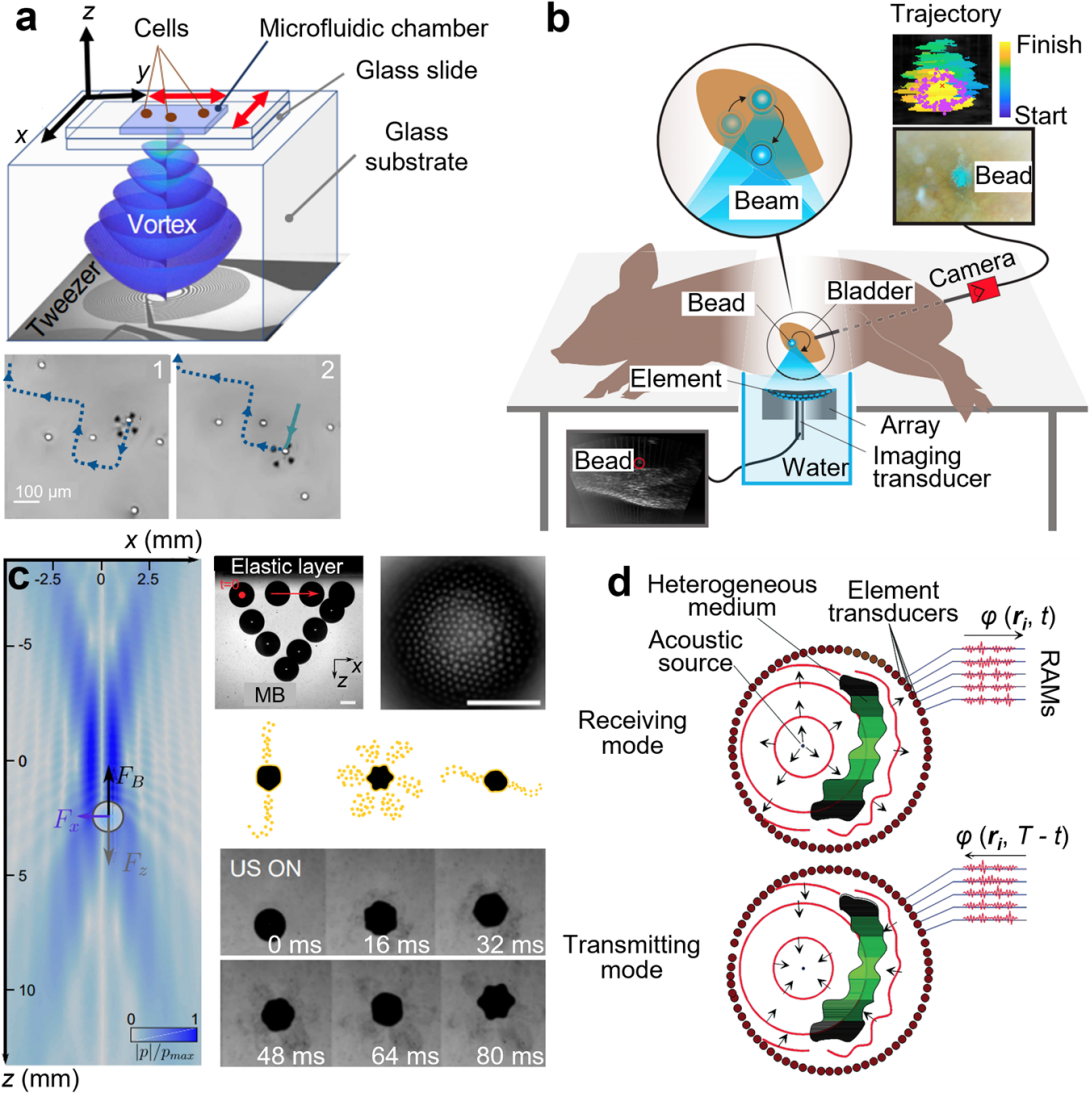
Acoustic technologies for in vitro and in vivo manipulation. **a** Acoustic vortex-based tweezers enable the selective manipulation of individual cells and particles within a microchannel. **b** Vortex-based tweezers have been used to controllably move a 3-mm glass sphere inside a pig’s bladder to mimic the repositioning of a kidney stone. **c** Vortex-based tweezers have also been used to trap and maneuverer microbubbles in 3D through materials that mimic biological tissues. In addition, nanoparticle-coated microbubbles can release their payload in one or several directions, offering an approach for highly directional drug delivery. **d** Time-reversal acoustics offer an approach for the focusing of acoustic energy within complex biological environments. Transducers are used to record the acoustic field emanating from an acoustic source and then send back a time-reversed version of that same signal. Part **a** adapted from ref. ^45^, CC BY 4.0 (https://creativecommons.org/licenses/by/4.0/). Part **b** adapted with permission from ref. ^47^, National Academy of Science. Part **c** adapted with permission from ref. ^48^, CC BY 4.0 (https://creativecommons.org/licenses/by/4.0/). Part **d** used with permission of Society of Exploration Geophysicists (SEG), from *Time-reversal acoustics in complex environments* (10.1190/1.2215356), Mathias Fink, volume 71, issue 4 (2006) permission conveyed through Copyright Clearance Center, Inc.

In addition to acoustic holograms, a new acoustic tweezing technique based on acoustical vortices, which are tightly focused helical acoustic waves, has recently been demonstrated to selectively manipulate individual cells within a microchamber in a contact-free manner^45^. Previously demonstrated acoustic tweezers utilize a grid-like pattern of trapping sites, and as a result, it is challenging to manipulate individual particles without disturbing nearby cells. On the other hand, acoustic vortex-based tweezers generate a highly focused, single trapping site, enabling the selective capture, positioning, and release of cells within the microchamber (Fig. 5a). Compared to its more established, widely used optical counterpart, optical tweezers, acoustic vortex-based tweezers can generate forces that are one order of magnitude higher (~200 pN) while applying an acoustic wave power that is one order of magnitude lower (~2 mW). This highly biocompatible approach for manipulating cells can enable new possibilities for probing cellular communication, investigating mechanotransduction mechanisms, and precisely positioning cells to fabricate complex tissue structures.

To foster the development of more practical acoustic-enabled 3D tissue engineering, spheroids, or organoids technologies, it will be beneficial to demonstrate the simultaneous patterning of cells and nanoscale components like matrix fibers. In order to manipulate nanoparticles, it is necessary to increase the magnitude of the acoustic forces. However, increasing the magnitude of the acoustic forces usually introduces issues such as unwanted acoustic streaming and elevated temperatures. Potential solutions include using more efficient acoustic transducers and integrating secondary-focused ultrasound technologies for modifying the cellular microenvironment. Furthermore, to improve accessibility to acoustic technologies for tissue engineering, it is necessary to transition from customized prototypes and in-house research instruments to commercialized products with widely applicable standard operating procedures. For example, more precise and functional demonstrations in the large-scale manufacturing of organoids by acoustic waves for patterning brain organoids or other organoids are critical for realizing truly transformative biomedical applications. One acoustic approach that may provide a pathway to manufacturing more complex organoids is a recently developed hexagonal acoustofluidic platform^46^. By using three pairs of acoustic transducers, the researchers created a hexagonal acoustic field within a commonly used cell culture dish, removing the need for highly specialized equipment. By adjusting the phase and amplitude applied to each transducer, organoids within the chamber could be rotated, transported, and fused with one another in a contact-free manner. To demonstrate the potential of this platform, the researchers fused human forebrain organoids and human midbrain organoids and demonstrated that these complex assembloids exhibited neuron higher firing rates and higher synchrony than either of the individual organoids. This technology represents a promising and straightforward approach to fabricating complex assembloids and may be particularly valuable for developmental biology and drug screening research.

### In vivo acoustic manipulation: non-invasive acoustic surgical revolution

The motivation for developing non-invasive manipulation techniques within the human body stems from the fact that nearly one in six surgical patients experience complications of some kind during surgery. In the United States, over 50 million inpatient surgeries are performed each year^47^, leading to a tremendous demand for the development of non-invasive surgical alternatives. Acoustic manipulation offers one potential approach to non-invasively move solid objects within the body. If successful, in vivo acoustic manipulation could have many medical applications including the steering of catheters, removing inaccessible or inoperable foreign bodies, or repositioning urinary stones.

Recently, Ghanem et al. developed a steerable, vortex-based acoustic trapping beam to lift and move objects within a living body (Fig. 5b)^47^. The steerable acoustic beam was generated by a 256-element phased array of transducers, which enabled objects to be trapped at the beam’s center and moved in two dimensions transverse to the beam axis. This represents a significant advance over previously developed in vivo acoustic manipulation strategies, which can only push objects away from the transducer for applications such as kidney stone expulsion. The acoustic beam produced by Ghanem et al. was used to controllably move a 3-mm glass sphere inserted through the ureter into a pig’s bladder to mimic a kidney stone. The sphere could be controllably moved in three dimensions, and after ultrasound exposure, no histological evidence of injury was observed in the pigs, demonstrating the non-invasive nature of the technique. This work paves the way for the future development of more advanced medical applications and lays the groundwork for translating in vivo acoustic manipulation to human patients. In the coming decade, could we see the development of more advanced in vivo acoustic manipulation technologies that will enable contact-free camera steering, potentially replacing many of the bulky, wired devices that are currently used to perform endoscopies?

In addition to the in vivo manipulation of millimeter-sized solid objects, acoustic beams also show great promise in the three-dimensional trapping and manipulation of microparticles, such as microbubbles, within the human body. In recent years, microbubbles have been increasingly used for biomedical applications such as targeted drug delivery and as contrast agents for high-resolution imaging. The ability to selectively trap and manipulate microparticles would enable researchers to design more complex and precise drug delivery strategies, and unlock new applications, such as in vivo tissue engineering. Recently, Baresch et al. demonstrated the potential for using acoustic beams to manipulate microbubbles in vivo (Fig. 5c)^48^. A vortex acoustic beam was used to control microbubbles in 3D through materials that mimicked biological tissues, including agarose gels of varying thicknesses and opacities. The nanoparticle-coated microbubbles could be held in place and activated by an independent acoustic source, where they would release their payload in a directed manner. Lo et al. also used similar vortex-based tweezers to demonstrate the trapping of microbubbles within the capillary of a mouse, offering a biocompatible, contact-free approach for concentrating low-dose drug carriers^49^. These demonstrations of the in vivo manipulation of microscale non-solid objects represent a significant advance in acoustic manipulation technologies, which had previously only shown the ability to trap solid objects.

Most in vivo acoustic manipulation technologies thus far have shown the ability to manipulate only spherical objects. However, objects for practical applications will rarely be perfectly spherical, resulting in spinning and slipping from the acoustic trap. For in vivo technologies to find real-world clinical applications, it will be necessary to demonstrate the reliable trapping and maneuvering of non-spherical, irregularly shaped objects. Although the in vivo manipulation of irregularly shaped objects represents a significant challenge, recent demonstrations in the field of acoustic levitation may offer inspiration. An “acoustic lock” mechanism, based on twin acoustic traps, has recently been demonstrated to trap non-spherical objects in the air^50^. In the future, perhaps similar twin acoustic traps can be utilized to trap non-spherical objects within the human body. An additional challenge for in vivo acoustic manipulation results from the complex internal environments with inconsistent impedance distributions found in the human body, leading to wave scattering and reducing acoustic trapping efficiency. One way to improve the performance of acoustic trapping in complex environments that have not yet been thoroughly explored is using time-reversal acoustics (Fig. 5d)^51^, which enables more precise focusing of acoustic energy within the body.

### Perspective, challenges, and future directions

Acoustic technologies for life sciences and medicine have evolved rapidly over the past decade. We strongly believe that the four research areas discussed in this perspective article will drive the future development of acoustic technologies over the next decade. In each of the four areas discussed above, we expect (1) Improvements to focus acoustic energy with increased spatial and temporal resolution will enable more detailed studies of mechanosensitive ion channels. We expect continued development in this area and envision the development of non-invasive acoustic stimulation tools for Piezo channel-based drug discovery and delivery, neural activation and inactivation, and cancer therapy. (2) A focus on commercial prototype development will bring acoustofluidic point-of-care technologies from specialized research labs to hospitals, laboratories, and mobile clinics. Over the coming decade, we expect to see a shift from the current “chip-in-a-lab” approach of technology demonstration to the fully functional, portable “lab-on-a-chip” approach promised by these exciting technologies. (3) As the throughput of acoustic technologies in biomedical engineering applications is improved, we predict more widespread commercial and clinical adoption of acoustic technologies. Similar to the widespread proliferation of filtration technologies in pharmaceutical manufacturing, we envision the rapid expansion of acoustic technologies to fabricate tissue constructs, cell spheroids, and organoids for both research and pharmaceutical applications. (4) As the ability to focus acoustic energy within the human body improves, we expect to see practical applications of in vivo acoustic manipulation, such as the contact-free steering of cameras or microrobots, emerge. The exciting proof-of-concept demonstrations of in vivo acoustic manipulation outlined above establish a promising foundation for developing future translational surgical tools. While many advances have been made in developing next-generation acoustic technologies, addressing these key challenges is critical for ensuring that advances in acoustics research will provide promising solutions for future life sciences and medical applications.
